# Electrophysiological Responses to Rapidly-Presented Affective Stimuli Predict Individual Differences in Subsequent Attention

**DOI:** 10.1523/ENEURO.0285-21.2021

**Published:** 2022-02-10

**Authors:** Ha Neul Song, Sewon Oh, Sang Ah Lee

**Affiliations:** 1 Department of Brain and Cognitive Sciences, Seoul National University, Seoul 08826, Republic of Korea; 2 Department of Bio and Brain Engineering, Korea Advanced Institute of Science and Technology, Daejeon 34141, Republic of Korea; 3 Department of Psychology, University of South Carolina, Columbia, SC 29208

**Keywords:** attention, EEG, emotion, individual differences, skin conductance response

## Abstract

We are constantly surrounded by a dynamically changing perceptual landscape that can influence our behavior even without our full conscious awareness. Emotional processing can have effects on subsequent attention, but there are mixed findings on whether it induces attentional enhancement or interference. The present study used a new multimodal approach to explain and predict such attentional effects based on individual differences in response to emotional stimuli. We briefly presented affective pictures (neutral, positive, erotic, mutilation, and horror categories) for 80 ms, immediately followed by a cued flanker task that was unrelated to the pictures. Event-related potentials (ERPs), skin conductance response (SCR), and reaction time (RT) were measured for each participant. We found that, in general, affective pictures induced higher electrophysiological responses compared with neutral pictures [P300 and late positive potential (LPP) in the erotic condition; P300, LPP, and SCR in the horror condition]. In particular, individuals who showed a strong ERP response to the pictures were impeded in the erotic condition (only P300) and facilitated in the horror condition (both P300 and LPP). Those who did not show a significant ERP or SCR response to the pictures were facilitated in the erotic condition and impeded in the horror condition. Furthermore, it was possible to classify the direction of the attentional effect from the participants’ P300, LPP, and SCR responses. These results demonstrate that underlying individual differences in emotional processing must be considered in understanding and predicting the effects of emotions on attention and cognition.

## Significance Statement

Automatic influence of emotions on subsequent attention may be adaptive for fast behavioral response to environmental stimuli. The majority of past studies have claimed that pleasant emotions facilitate subsequent attention and that unpleasant emotions impede it. However, several studies directly contradicted such findings by reporting opposite effects, with pleasant pictures impeding attention and unpleasant pictures facilitating it. Our results resolve this discrepancy in the existing literature by showing that depending on how weakly or strongly someone responds to emotional stimuli (erotic and horror categories), they may be either facilitated or distracted in their subsequent attention. Furthermore, we were able to accurately classify the direction of this attentional effect using their event-related potential (ERP) and skin conductance response (SCR) to the pictures.

## Introduction

Recently, research in brain and cognitive sciences has started to interface closely with applications for improving cognition and mental health. One way in which such tools are used is for personal emotion monitoring and regulation. Most current technology, however, requires that users explicitly recognize their internal states (e.g., through self-report). Yet, in everyday life, people are constantly bombarded with rapidly changing perceptual stimuli that may trigger brain processes which can influence them even while they are engaged in other tasks (Halgren, 1992; Compton, 2003; Vuilleumier, 2005; Vuilleumier and Driver, 2007; Bradley, 2009; Moser et al., 2010; Pourtois et al., 2013; LeBlanc et al., 2015). For example, after passing an animal on the side of the road, a driver may become momentarily susceptible to missing a turn or getting into an accident without fully being aware of what he saw. Subsequent attentional effects induced by emotional stimuli can vary depending on the individual; in the situation described above, some people may become more alert while others get distracted after passing the scene. Although individual differences in emotional processing have been studied extensively, there is a lack of understanding on how such differences influence attention (Gohm and Clore, 2000; Hamann and Canli, 2004; Mardaga et al., 2006; Zhang and Zhou, 2014; Matusz et al., 2015).

Emotional processing consists of detecting and responding to (e.g., via arousal and regulation) emotionally significant perceptual stimuli and can have multiple pathways by which it affects subsequent attention (Pourtois et al., 2013). Because such processes can happen quickly, their quantitative measurement requires high temporal resolution. Event-related potentials (ERPs) in response to emotional stimuli provide simple and fast markers of cortical activity (Hajcak et al., 2013b) that can be easily acquired using a variety of EEG systems. According to previous studies, P300 (positive potential occuring about 300ms after stimulus onset) amplitude correlates with perceived emotional significance and late positive potential (LPP) amplitude with emotion regulation (Johnston et al., 1986; Cuthbert et al., 2000; Foti and Hajcak, 2008; Hajcak et al., 2010; Hajcak and Foti, 2020). In addition, skin conductance response (SCR), which indicates activity of the sympathetic nervous system and is associated with hypothalamic arousal, has a slower progression and is longer-lasting compared with ERPs (Critchley et al., 2000; Cuthbert et al., 2000). As different aspects of emotional processing are reflected in each physiological marker, a multimodal approach using ERP and SCR may enhance our ability to explain and predict the cognitive effects of emotional processing at the individual level.

Given that fast processing of emotions are adaptive mechanisms for subsequent behavioral responses, it seems reasonable for even quickly presented emotional stimuli to modulate attention; however, there have been mixed findings on the direction of such effects (Schmeichel, 2007; Bocanegra and Zeelenberg, 2009, 2011; Ortner et al., 2013). Furthermore, while electrophysiological correlates of attention on emotional stimuli themselves have been well-documented (e.g., N2, EPN, LPP), their relevance to attention on an unrelated task has not yet been characterized extensively (Krolak-Salmon et al., 2001; Pourtois et al., 2004; Sabatinelli et al., 2007; Olofsson et al., 2008; Wiens et al., 2011; Hajcak et al., 2013a). Some studies reported that pleasant emotional stimuli facilitate subsequent attention and that unpleasant stimuli impede it (Eastwood et al., 2003; Wadlinger and Isaacowitz, 2006; Friedman and Förster, 2010; LeBlanc et al., 2015). However, others have yielded contrary results. In one study, images of fearful faces enhanced, rather than decreased, performance in a perceptual attention task (Phelps et al., 2006). Another study reported that briefly-presented sexual stimuli decreased performance in a dot detection task; interestingly, the magnitude of this effect was correlated with self-reports of eroticism (Prause et al., 2008).

One overlooked factor is that individual differences may not only explain the magnitude of such effects but also their direction (facilitation vs impediment). Because the same emotional stimulus can elicit varied responses across individuals according to their personal characteristics or experiences, the current study investigated individual differences in the interaction between emotional processing and attention and hypothesized that people whose attention is facilitated by affective pictures would show dissociable physiological responses from those who are impeded by it. Through this investigation, we aimed not only to provide insight into the mechanisms underlying the interaction between emotion and cognition but to also improve personalization of neurotechnology and its real-world applicability.

To simulate situations in which attention is automatically influenced by rapid emotional processing, we briefly presented participants with affective pictures before teach trial of a cued flanker task (Fan et al., 2002, 2005). Neutral, positive, erotic, mutilation, and horror picture stimuli were used to elicit a variety of potentially emotion-dependent effects. To explore individual differences, we divided people into two groups based on whether they were facilitated or impeded by certain picture categories and compared their ERP and SCR measures. Finally, we tested whether these physiological markers can accurately classify and predict attentional effects at the individual level.

## Materials and Methods

### Participants

Participants were thirty-one university students (19 males, mean age 24.77, SD = 3.74) recruited from the Daejeon area. All participants were right-handed and had normal or corrected vision. Data from all participants were included in the group analysis involving SCR and reaction time (RT). Data from five participants were excluded from the analysis of EEG data due to a failure to acquire usable data (disrupted connection or interrupted testing session), resulting in a final sample size of 26 (14 males). All participants’ anxiety and depression scores were measured via Beck Anxiety Inventory (BAI) and Beck Depression Inventory-II (BDI-II); no participants were found to have severe anxiety or depression (Beck et al., 1988, 1996).

Supporting data were collected from three separate independent samples: picture stimuli valence/arousal rating (*n* = 10, mean age = 23.20, SD =* *2.44), picture awareness and memory test (*n* = 17, mean age = 22.35, SD =* *4.27), and a partial replication of the findings using a 32-channel wired EEG system (eight males, mean age = 28.35, SD =* *4.19).

### Materials and procedures

**Figure 1.**
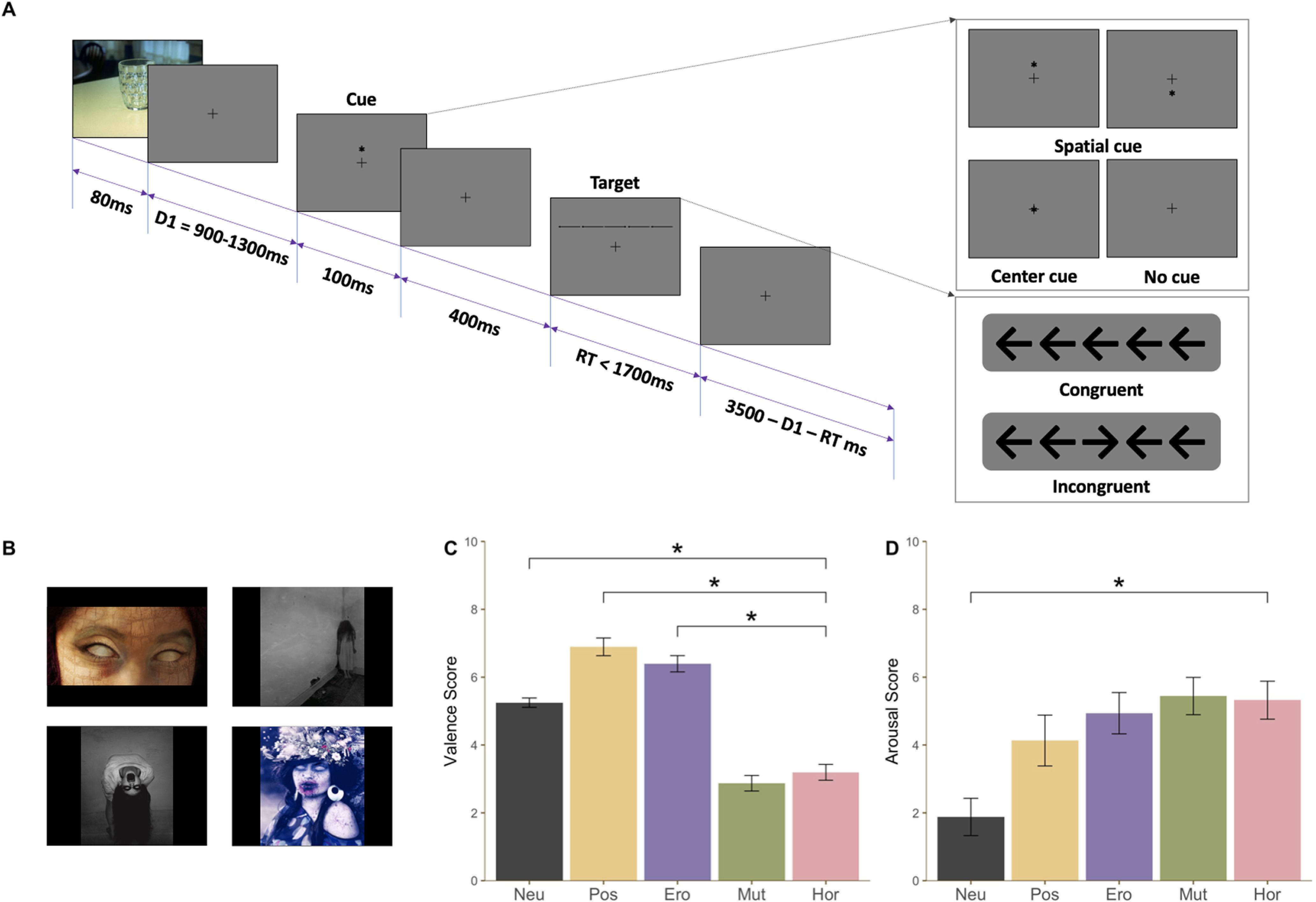
***A***, Task sequence. A picture (neutral, positive, erotic, mutilation, or horror) was presented for 80 ms to induce emotional processing before each trial of the cued ANT. When a row of five arrows appeared, participants were asked to indicate the direction of the center arrow (target) as quickly as possible. RT was measured. ***B***, Examples of horror pictures. A total of 48 horror pictures from a commercially usable free web source were used (other picture categories were taken from the IAPS database). ***C***, ***D***, Validation of valence (***C***) and arousal (***D***) ratings for the non-IAPS horror pictures. Ten subjects were separately recruited to rate the stimulus set on their valence and arousal. Valence ratings for pictures in the horror category were lower than the ratings for neutral, positive, and erotic pictures but not different from mutilation pictures (***C***). Arousal ratings for horror pictures were higher than the ratings for neutral pictures but not different from mutilation pictures (***D***). Black asterisks indicate corrected *p*s < 0.05 for nonparametric paired tests.

We aimed to induce rapid emotional processing via brief presentations of visual scenes immediately followed by a trial of a cued flanker task [attention network task (ANT); Fan et al., 2002, 2005]. On each trial, the affective picture was presented for 80 ms (for more information, see Figs. 1*A*), followed by a randomized fixation period between 900 and 1300 ms long. For cued trials, an asterisk appeared for 100 ms (either above, center, or below the fixation point) and, after 400 ms of fixation, the target was presented. The ANT task, designed to engage multiple attentional mechanisms, employed a center asterisk (center cue) to give participants temporal information about the target presentation, and the placement of the asterisk above or below the center fixation point (spatial cue) additionally provided information about where the target will appear. The target was the center arrow of a row of five arrows; on congruent trials, the flanker arrows were consistent with the direction of the target arrow, and on incongruent trials, they pointed in the opposite direction. Participants were asked to indicate the direction of the target arrow as quickly as possible; if they did not respond within 1700 ms, the fixation period for the next trial started automatically. RT on each trial was recorded and log transformed to minimize skewed distribution of each participant’s data. After the participants made a response, the arrows disappeared and a fixation period followed.

Before the start of the test session, participants were given 20 practice trials to familiarize themselves with the task flow. The main task consisted of 10 blocks of 24 trials each. Two-minute-long breaks were given between the blocks. Each emotion condition (neutral, positive, erotic, mutilation, and horror) was tested across two blocks, once in the first half of the session and another in the second half. The order of the blocks within each half was randomized, with the restriction that the same emotion condition block did not appear in succession (i.e., fifth and sixth blocks were not the same). During the task, EEG, SCR (right-side two fingers in hardware), and RT (button press with left hand) were recorded simultaneously.

With the exception of the pictures in the horror condition, all pictures were selected from the International Affective Picture System (IAPS), a commonly used image database for emotion research containing the standardized valence score and situational category of each picture (Lang et al., 2008). For the neutral condition, pictures in the median 20% of IAPS valence scores were selected, excluding those containing images of people, weapons, cigarettes, and food, to avoid socially biased effects. From pictures with top 20% valence scores in the IAPS data base, those of intimately engaged heterosexual couples were selected for the erotic condition, and those excluding sexual content were selected for the positive condition. The mutilation condition consisted of images of bodily damage/harm selected based on the IAPS picture descriptions. Since the fear-inducing pictures included in the IAPS data base were inadequate to be categorized as “horror,” the horror condition pictures were selected from a commercially usable free web source. The horror pictures’ comparability to other conditions was confirmed before the main task (Fig. 1*B*). A separate group of 10 participants rated the valence and arousal of all of the pictures in our stimulus set, after each picture was presented for 3 s on a computer monitor in front of them. Altogether, 240 pictures were used, 48 from each emotion condition.

### SCR data acquisition

To detect the release of sweat due to a change in the arousal state (Montagu and Coles, 1966), SCR (galvanic skin response) was measured using the Gazepoint biometrics package and software, with a constant voltage coupler (5 V) and a 60-Hz sampling rate. Participants put their right index and middle fingers into the biometric hardware and were instructed to pull out their fingers between task blocks to prevent the physiological response from saturation. To calculate SCR for each picture, a high-pass FIR filter of 0.05 Hz was applied (MATLAB) to the entire time series; then, maximum change in SCR was extracted from the baseline (average over the 500-ms fixation period preceding picture onset) to the test trial (from picture onset to 500 ms before the next trial). The data were log transformed to minimize skewness and averaged for each block (for multimodal classification) and each emotional condition (for the remainder of the analysis).

### EEG data acquisition and processing

Participants’ EEG signal was recorded using the gel-type 32-channel wireless Emotiv EPOC Flex that adheres to the 10–20 system, a standard method for electrode placement. The data were preprocessed through average re-referencing and bandpass filtering between 0.1 and 30 Hz using EEGLAB on MATLAB (Delorme and Makeig, 2004). Based on the picture presentation at 0 ms, ERP epochs were selected from –100 to 1000 ms. Baseline (from –100 to 0 ms) correction was applied in each epoch. Epochs containing ocular artifacts (identified through Infomax ICA) or signals with an absolute value higher than 100 μV were omitted from the analysis (Delorme et al., 2007). Three channels (Fz, Cz, and Pz) were selected for ERP component analysis (Stormark et al., 1995; Cuthbert et al., 1998; Codispoti et al., 2006; Yen et al., 2010). P300 and LPP amplitudes were calculated using the mean voltage between 250 and 350 ms and between 500 and 800 ms, respectively; these time-points were chosen based on previous literature (Lu et al., 2011; Zhang and Zhou, 2014; Zhao et al., 2018; Maffei et al., 2021) and our study design in which the ANT began at least 900  ms after picture onset. To test for the effects of emotional stimuli, the three types of responses (RT, SCR, ERP) in the four emotion conditions (positive, erotic, mutilation, and horror) were compared with those in the neutral condition (see below for a description of notation) (Schupp et al., 2003, 2006a,b). Bonferroni correction was applied to the *p* value of E_modality, emotion_ based on the number of multiple comparisons following the repeated-measures ANOVA. The Greenhouse-Geisser correction was applied for violations of sphericity (adjusted degrees of freedom provided).

$$E_{modality,\;emotion}=\;R_{modality,\;emotion}–\;R_{modality,\;neutra\mathrm{l,}}$$

*E*: Effect of affective stimuli compared with the neutral condition;

*R*: response value in each modality and condition;

*modality*: RT, SCR, or ERP;

*emotion*: positive, erotic, mutilation, or horror.

### Prediction of facilitation versus impediment of attention

In the conditions which resulted in significant emotional effects on SCR and ERP, the participants were divided into two groups based on whether they were facilitated (E_RT, emotion_ < 0) or impeded (E_RT, emotion_ > 0). A support vector machine (SVM) was used to classify the subjects, based on their ERP (unimodal) or both ERP and SCR (multimodal), to predict whether RT in the emotion condition is faster or slower than that in the neutral condition (Noble, 2006). Block-averaged values were used for each variable. In each condition, prediction and accuracy and area under the receiver operating characteristic (ROC) curve (AUC) were calculated using a SVM with 10-fold cross-validation.

## Results

### Behavioral performance

Before the main experiment, we compared the valence and arousal ratings of the picture stimuli across all conditions; this was particularly relevant with respect to the horror condition, which was not a part of the IAPS. Non-parametric comparisons (Friedman’s test) revealed a main effect of the emotion condition for both valence ratings (χ^2^ = 38.000, *p* < 0.001; Fig. 1*C*) and arousal ratings (χ^2^ = 29.760, *p* < 0.001; Fig. 1*D*). As expected, *post hoc* Wilcoxon signed-rank tests showed that horror pictures were rated significantly lower in valence than neutral, positive, and erotic pictures but not differently from mutilation pictures. For arousal ratings, the horror condition was significantly higher than the neutral condition but not different from other emotion conditions (Table 1).

**Table 1:** Statistical table 1

#	Figure	Description	Data structure	Type of test	Statistical values	Significance	Effect size
1	1*D*, left	Valence	Normality not assumed	One-way Friedman’s test	χ^2^ = 38.000	*p* < 0.001	-
2	1*D*, left	Valence (neu vs pos)	Normality not assumed	*Post hoc* Wilcoxon signed-rank test	Z = −2.803	*p* corrected = 0.051	*r* = −0.886
3		Valence (neu vs ero)			Z = −2.803	*p* corrected = 0.051	*r* = −0.886
4		Valence (neu vs mut)			Z = 2.803	*p* corrected = 0.046	*r* = 0.886
5		Valence (neu vs hor)			Z = 2.803	*p* corrected = 0.041	*r* = 0.886
6		Valence (pos vs ero)			Z = 1.886	*p* corrected = 0.119	*r* = 0.596
7		Valence (pos vs mut)			Z = 2.803	*p* corrected = 0.035	*r* = 0.886
8		Valence (pos vs hor)			Z = 2.803	*p* corrected = 0.030	*r* = 0.886
9		Valence (ero vs mut)			Z = 2.803	*p* corrected = 0.025	*r* = 0.886
10		Valence (ero vs hor)			Z = 2.803	*p* corrected = 0.020	*r* = 0.886
11		Valence (mut vs hor)			Z = −1.580	*p* corrected = 0.119	*r* = −0.500
12	1*D*, right	Arousal	Normality not assumed	One-way Friedman’s test	χ^2^ = 29.760	*p* < 0.001	-
13	1*D*, right	Arousal (neu vs pos)	Normality not assumed	*Post hoc* Wilcoxon signed-rank test	Z = −2.803	*p* corrected = 0.051	*r* = −0.886
14		Arousal (neu vs ero)			Z = −2.803	*p* corrected = 0.051	*r* = −0.886
15		Arousal (neu vs mut)			Z = −2.803	*p* corrected = 0.046	*r* = −0.886
16		Arousal (neu vs hor)			Z = −2.803	*p* corrected = 0.041	*r* = −0.886
17		Arousal (pos vs ero)			Z = −2.192	*p* corrected = 0.124	*r* = −0.693
18		Arousal (pos vs mut)			Z = −2.497	*p* corrected = 0.075	*r* = −0.790
19		Arousal (pos vs hor)			Z = −2.244	*p* corrected = 0.124	*r* = −0.710
20		Arousal (ero vs mut)			Z = −1.478	*p* corrected = 0.418	*r* = −0.467
21		Arousal (ero vs hor)			Z = −1.172	*p* corrected = 0.482	*r* = −0.371
22		Arousal (mut vs hor)			Z = 0.459	*p* corrected = 0.647	*r* = 0.145

**Figure 2.**
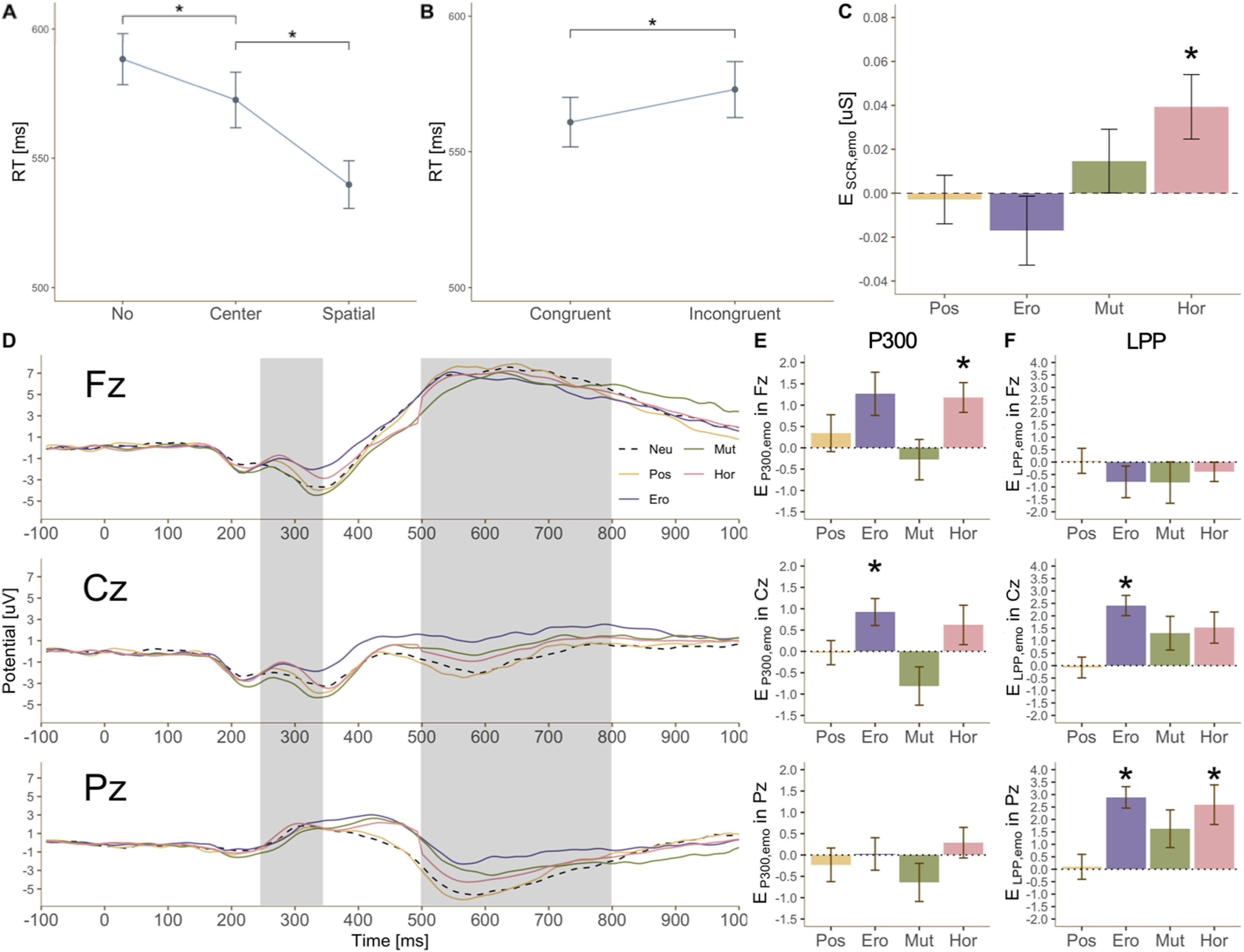
***A***, Behavioral performance across cue types. RT after the spatial cue was faster than that after the center cue; both were faster than having no cue at all. ***B***, Behavioral performance across target types. RT for the congruent target was faster than RT for the incongruent target. ***C***, SCR difference scores *E_SCR, emotion_* across emotion conditions. The dotted line indicates SCR in the neutral condition. SCR in the horror condition was higher than in the neutral condition. ***D***, ERP across emotion conditions after picture presentation in channels Fz, Cz, and Pz. Dotted and colored lines indicate ERPs, with the picture presented at time = 0 ms. P300 and LPP amplitudes were averaged between 250 and 350 ms and between 500 and 800 ms, respectively. ***E***, P300 difference scores *E_P300, emotion_* across emotion conditions. From the top to bottom, graphs show *E_P300, emotion_* in channels Fz, Cz, and Pz. Dotted lines indicate P300 in the neutral condition. P300 amplitudes in the horror condition (channel Fz) and erotic condition (channel Cz) were higher than the neutral condition. ***F***, LPP difference score *E_LPP, emotion_* across emotion conditions. From the top to bottom, graphs show *E_LPP, emotion_* in channels Fz, Cz, and Pz. Dotted lines indicate LPP in the neutral condition. LPP amplitudes in the erotic condition (channels Cz and Pz) and horror condition (channel Pz) were higher than in the neutral condition. Black asterisks indicate corrected *p*s < 0.05 for paired *t* tests or one-sample *t* tests.

A three-way repeated measures ANOVA including the emotion condition (neutral, positive, erotic, mutilation, and horror), cue condition (spatial, center, and no), and target condition (congruent and incongruent) was conducted to analyze their effects on RT and make sure that the cue and target in our modified ANT worked properly. There were main effects of the cue and target conditions on RT [*F*_(1.601,48.025)_ = 49.605, *p* < 0.001, η_p_^2^ = 0.213 (Fig. 2*A*); *F*_(1,30)_ = 8.109, *p* = 0.008, η_p_^2^ = 0.623 (Fig. 2*B*)]. For *post hoc* pairwise *t* tests, RT after the spatial cue was faster than that after both the center cue (*t*_(30)_ = −5.871, *p*_corrected_ < 0.001, *d* = −1.054) and no cue (*t*_(30)_ = −8.659, *p*_corrected_ < 0.001, *d* = −1.555). RT following the center cue was faster than no cue (*t*_(30)_ = −4.517, *p*_corrected_ < 0.001, *d* = 0.811). RT for the congruent target was also faster than that for the incongruent target (*t*_(30)_ = −2.826, *p*_corrected_ = 0.008, *d* = −0.508). There was a main effect of emotion but no significant results in the *post hoc* pairwise comparisons (*F*_(4,120)_ = 2.839, *p* = 0.027, η_p_^2^ = 0.086). The results showed that participants were able to correctly perform the ANT using the cue information and the congruency of arrows.

To test whether RT in each of the positive, erotic, mutilation, and horror conditions were different from that in the neutral condition, a one-sample *t* test with 0 was conducted on the RT difference score *E_RT, emotion_* in each emotion category. There were no significant differences (Table 2).

**Table 2:** Statistical table 2

#	Figure	Description	Data structure	Type of test	Statistical values	Significance	Effect size
1	2*A,B*	RT (cue)	Assumed normal	Three-way repeated measures ANOVA	*F*_(1.601,48.025)_ = 49.605	*p* < 0.001	η_p_^2^ = 0.213
2		RT (target)			*F*_(1,30)_ = 8.109	*p* = 0.008	η_p_^2^ = 0.623
3		RT (emotion)			*F*_(4,120)_ = 2.839	*p* = 0.027	η_p_^2^ = 0.086
4		RT (cue × target)			*F*_(1.654,49.629)_ = 1.756	*p* = 0.188	η_p_^2^ = 0.055
5		RT (cue × emotion)			*F*_(8,240)_ = 0.843	*p* = 0.565	η_p_^2^ = 0.027
6		RT (target × emotion)			*F*_(4,120)_ = 1.405	*p* = 0.237	η_p_^2^ = 0.045
7		RT (cue × target × emotion)			*F*_(5.774,173.213)_ = 0.873	*p* = 0.513	η_p_^2^ = 0.028
8	2*A*	Cue (no vs left)	Assumed normal	*Post hoc* pairwise *t* test	*t*_(30)_ = 4.517	*p* corrected < 0.001	*d* = 0.811
9		Cue (no vs spatial)			*t*_(30)_ = 8.659	*p* corrected < 0.001	*d* = 1.555
10		Cue (left vs spatial)			*t*_(30)_ = 5.871	*p* corrected < 0.001	*d* = 1.054
11	2*B*	Target (congruent vs incongruent)	Assumed normal	*Post hoc* pairwise *t* test	*t*_(30)_ = −2.826	*p* = 0.008	*d* = −0.508
12	-	E _RT, pos_	Assumed normal	One-sample *t* test	*t*_(30)_ = −1.985	*p* corrected = 0.169	*d* = −0.356
13		E _RT, ero_			*t*_(30)_ = −1.836	*p* corrected = 0.169	*d* = −0.330
14		E _RT, mut_			*t*_(30)_ = −2.216	*p* corrected = 0.138	*d* = −0.398
15		E _RT, hor_			*t*_(30)_ = −0.348	*p* corrected = 0.730	*d* = −0.063
16	-	E _RT, emotion_	Assumed normal	One-way repeated measures ANOVA	*F*_(3,90)_ = 2.546	*p* = 0.061	η_p_^2^ = 0.078
17	2*C*	E _SCR, pos_	Assumed normal	One-sample *t* test	*t*_(30)_ = −0.261	*p* corrected = 0.796	*d* = −0.047
18		E _SCR, ero_			*t*_(30)_ = −1.087	*p* corrected = 0.857	*d* = −0.195
19		E _SCR, mut_			*t*_(30)_ = 1.007	*p* corrected = 0.857	*d* = 0.181
20		E _SCR, hor_			*t*_(30)_ = 2.675	*p* corrected = 0.048	*d* = 0.481
21	2*C*	E _SCR, emotion_	Assumed normal	One-way repeated measures ANOVA	*F*_(3,90)_ = 4.955	*p* = 0.003	η_p_^2^ = 0.142
22	2*C*	E _SCR, emotion_ (pos vs hor)	Assumed normal	*Post hoc* pairwise *t* test	*t*_(30)_ = −2.813	*p* corrected = 0.043	*d* = −0.505
23		E _SCR, emotion_ (ero vs hor)			*t*_(30)_ = −3.377	*p* corrected = 0.012	*d* = −0.607
24	2*E*, top	E _P300, pos_ in Fz	Assumed normal	One-sample *t* test	*t*_(25)_ = 0.792	*p* corrected = 0.871	*d* = 0.154
25		E _P300, ero_ in Fz			*t*_(25)_ = 2.4981	*p* corrected = 0.058	*d* = 0.490
26		E _P300, mut_ in Fz			*t*_(25)_ = −0.586	*p* corrected = 0.871	*d* = −0.115
27		E _P300, hor_ in Fz			*t*_(25)_ = 3.387	*p* corrected = 0.009	*d* = 0.664
28	2*E*, mid	E _P300, pos_ in Cz	Assumed normal	One-sample *t* test	*t*_(25)_ = −0.102	*p* corrected = 0.919	*d* = −0.020
29		E _P300, ero_ in Cz			*t*_(25)_ = 2.923	*p* corrected = 0.029	*d* = 0.573
30		E _P300, mut_ in Cz			*t*_(25)_ = −1.807	*p* corrected = 0.249	*d* = −0.354
31		E _P300, hor_ in Cz			*t*_(25)_ = 1.342	*p* corrected = 0.383	*d* = 0.263
32	2*E*, bottom	E _P300, pos_ in Pz	Assumed normal	One-sample *t* test	*t*_(25)_ = −0.583	*p* corrected = 1	*d* = −0.115
33		E _P300, ero_ in Pz			*t*_(25)_ = 0.064	*p* corrected = 1	*d* = 0.013
34		E _P300, mut_ in Pz			*t*_(25)_ = −1.436	*p* corrected = 0.653	*d* = −0.282
35		E _P300, hor_ in Pz			*t*_(25)_ = 0.806	*p* corrected = 1	*d* = 0.158
36	2*E*	E _P300, emotion_ (channel)	Assumed normal	Two-way repeated measures ANOVA	*F*_(1.568,39.193)_ = 1.757	*p* = 0.191	η_p_^2^ = 0.066
37		E _P300, emotion_ (emotion)			*F*_(3,75)_ = 9.065	*p* < 0.001	η_p_^2^ = 0.266
38		E _P300, emotion_ (channel × emotion)			*F*_(6,150)_ = 0.958	*p* = 0.456	η_p_^2^ = 0.037
39	2*E*	E _P300, emotion_ (pos vs ero)	Assumed normal	*Post hoc* pairwise *t* test	*t*_(25)_ = −2.926	*p* corrected = 0.029	*d* = −0.574
40		E _P300, emotion_ (ero vs mut)			*t*_(25)_ = 4.349	*p* corrected = 0.001	*d* = 0.853
41		E _P300, emotion_ (mut vs hor)			*t*_(25)_ = −4.074	*p* corrected = 0.002	*d* = −0.799
42	2*F*, top	E _LPP, pos_ in Fz	Assumed normal	One-sample *t* test	*t*_(25)_ = 0.099	*p* corrected = 0.922	*d* = 0.019
43		E _LPP, ero_ in Fz			*t*_(25)_ = −1.255	*p* corrected = 0.885	*d* = −0.246
44		E _LPP, mut_ in Fz			*t*_(25)_ = −0.992	*p* corrected = 0.961	*d* = −0.195
45		E _LPP, hor_ in Fz			*t*_(25)_ = −1.014	*p* corrected = 0.961	*d* = −0.199
46	2*F*, mid	E _LPP, pos_ in Cz	Assumed normal	One-sample *t* test	*t*_(25)_ = −0.180	*p* corrected = 0.859	*d* = −0.035
47		E _LPP, ero_ in Cz			*t*_(25)_ = 5.924	*p* corrected < 0.001	*d* = 1.162
48		E _LPP, mut_ in Cz			*t*_(25)_ = 1.926	*p* corrected = 0.131	*d* = 0.378
49		E _LPP, hor_ in Cz			*t*_(25)_ = 2.436	*p* corrected = 0.067	*d* = 0.478
50	2*F*, bottom	E _LPP, pos_ in Pz	Assumed normal	One-sample *t* test	*t*_(25)_ = 2.436	*p* corrected = 0.847	*d* = 0.038
51		E _LPP, ero_ in Pz			*t*_(25)_ = 6.708	*p* corrected < 0.001	*d* = 1.316
52		E _LPP, mut_ in Pz			*t*_(25)_ = 2.150	*p* corrected = 0.083	*d* = 0.422
53		E _LPP, hor_ in Pz			*t*_(25)_ = 3.258	*p* corrected = 0.010	*d* = 0.639
54	2*F*	E _LPP, emotion_ (channel)	Assumed normal	Two-way repeated measures ANOVA	*F*_(1.164,27.937)_ = 7.019	*p* = 0.010	η_p_^2^ = 0.226
55		E _LPP, emotion_ (emotion)			*F*_(3,72)_ = 5.598	*p* = 0.002	η_p_^2^ = 0.189
56		E _LPP, emotion_ (channel × emotion)			*F*_(2.753,66.070)_ = 3.998	*p* = 0.013	η_p_^2^ = 0.143
57	2*F*, mid	E _LPP, emotion_ (pos vs ero) in Cz	Assumed normal	*Post hoc* pairwise *t* test	*t*_(25)_ = −5.407	*p* corrected < 0.001	*d* = −1.060
58		E _LPP, emotion_ (pos vs hor) in Cz			*t*_(25)_ = −2.888	*p* corrected = 0.040	*d* = −0.566
59	2*F*, bottom	E _LPP, emotion_ (pos vs ero) in Pz	Assumed normal	*Post hoc* pairwise *t* test	*t*_(25)_ = −5.735	*p* corrected < 0.001	*d* = −1.125
60		E _LPP, emotion_ (pos vs hor) in Pz			*t*_(25)_ = −2.940	*p* corrected = 0.035	*d* = −0.577
61	3*C*, top left	E _P300, ero_ in Fz	Normality not assumed	One-sample Wilcoxon signed-rank test	Z = 1.782	*p* corrected = 0.075	*r* = 0.494
62		E _P300, ero_ in Cz			Z = −0.035	*p* corrected = 0.972	*r* = −0.010
63		E _P300, ero_ in Pz			Z = 0.315	*p* corrected = 1	*r* = 0.087
64	3*C*, top right	E _P300, ero_ in Fz	Normality not assumed	One-sample Wilcoxon signed-rank test	Z = 2.551	*p* corrected = 0.022	*r* = 0.708
65		E _P300, ero_ in Cz			Z = 3.180	*p* corrected = 0.003	*r* = 0.882
66		E _P300, ero_ in Pz			Z = 0.315	*p* corrected = 1	*r* = 0.087
67	3*C*, bottom left	E _LPP, ero_ in Fz	Normality not assumed	One-sample Wilcoxon signed-rank test	Z = 0.245	*p* corrected = 0.807	*r* = 0.068
68		E _LPP, ero_ in Cz			Z = 2.551	*p* corrected = 0.011	*r* = 0.708
69		E _LPP, ero_ in Pz			Z = 3.110	*p* corrected = 0.003	*r* = 0.863
70	3*C*, bottom right	E _LPP, ero_ in Fz	Normality not assumed	One-sample Wilcoxon signed-rank test	Z = −1.223	*p* corrected = 0.443	*r* = −0.339
71		E _LPP, ero_ in Cz			Z = 3.110	*p* corrected = 0.003	*r* = 0.863
72		E _LPP, ero_ in Pz			Z = 3.040	*p* corrected = 0.003	*r* = 0.843
73	3*D*, top left	E _P300, hor_ in Fz	Normality not assumed	One-sample Wilcoxon signed-rank test	Z = 2.341	*p* corrected = 0.038	*r* = 0.649
74		E _P300, hor_ in Cz			Z = 0.944	*p* corrected = 0.691	*r* = 0.262
75		E _P300, hor_ in Pz			Z = 1.572	*p* corrected = 0.232	*r* = 0.436
76	3*D*, top right	E _P300, hor_ in Fz	Normality not assumed	One-sample Wilcoxon signed-rank test	Z = 1.852	*p* corrected = 0.064	*r* = 0.514
77		E _P300, hor_ in Cz			Z = 0.804	*p* corrected = 0.691	*r* = 0.223
78		E _P300, hor_ in Pz			Z = −1.223	*p* corrected = 0.232	*r* = −0.339
79	3*D*, bottom left	E _LPP, hor_ in Fz	Normality not assumed	One-sample Wilcoxon signed-rank test	Z = 0.175	*p* corrected = 0.861	*r* = 0.049
80		E _LPP, hor_ in Cz			Z = 1.712	*p* corrected = 0.174	*r* = 0.475
81		E _LPP, hor_ in Pz			Z = 2.271	*p* corrected = 0.046	*r* = 0.630
82	3*D*, bottom right	E _LPP, hor_ in Fz	Normality not assumed	One-sample Wilcoxon signed-rank test	Z = −1.503	*p* corrected = 0.266	*r* = −0.417
83		E _LPP, hor_ in Cz			Z = 1.503	*p* corrected = 0.174	*r* = 0.417
84		E _LPP, hor_ in Pz			Z = 1.852	*p* corrected = 0.064	*r* = 0.514

### SCR

To test whether SCR in each of the positive, erotic, mutilation, and horror conditions was different from that in the neutral condition, a one-sample *t* test with 0 was conducted on the SCR difference score *E_SCR, emotion_* in each emotion category. SCR in the horror condition was higher than that in the neutral condition (*t*_(30)_ = 2.675, *p*_corrected_ = 0.048, *d* = 0.481; Fig. 2*C*). Furthermore, a one-way repeated measures ANOVA revealed a significant effect of the emotion condition on SCR (*F*_(3,90)_ = 4.955, *p* = 0.003, η_p_^2^ = 0.142), with *post hoc* pairwise *t* tests showing that *E_SCR, horror_* was higher than *E_SCR, positive_* and *E_SCR, erotic_* (Table 2). The results indicated that physiological arousal was significantly elicited in the horror condition.

### ERP response

To test whether P300 amplitude in each of the positive, erotic, mutilation, and horror conditions was different from that in the neutral condition, a one-sample *t* test with 0 was conducted on the P300 difference score *E_P300, emotion_* in each emotion condition and in each EEG channel (Fig. 2*E*). In channel Fz, P300 amplitude in the horror condition was higher than that in the neutral condition (*t*_(25)_ = 3.387, *p*_corrected_ = 0.009, *d* = 0.664). In channel Cz, P300 amplitude in the erotic condition was higher than that in the neutral condition (*t*_(25)_ = 2.923, *p*_corrected_ = 0.029, *d* = 0.573). To differentiate emotional effects on P300, a two-way repeated measures ANOVA including the emotion condition (positive, erotic, mutilation, and horror) and channel (Fz, Cz, and Pz) was performed. There was a main effect of emotion condition (*F*_(3,75)_ = 9.065, *p* < 0.001, η_p_^2^ = 0.266). Since there was no main effect of channel, the P300 amplitudes in channels Fz, Cz, and Pz were averaged for a *post hoc* pairwise *t* test. *E_P300, erotic_* was higher than *E_P300, positive_*, and both *E_P300, erotic_* and *E_P300, horror_* were higher than *E_P300, mutilation_* (Table 2). Increased P300 amplitudes for only the erotic and horror pictures may indicate that participants processed the emotional significance of these particular visual stimuli even after a brief presentation.

For LPP amplitudes, a one-sample *t* test against 0 was conducted on the LPP difference score *E_LPP, emotion_* in each emotion condition and channel (Fig. 2*F*). LPP in both the erotic and horror conditions was higher than that in the neutral condition in channel Pz (*t*_(25)_ = 6.708, *p*_corrected_ < 0.001, *d* = 1.316; *t*_(25)_ = 3.258, *p*_corrected_ = 0.010, *d* = 0.639), while only the erotic condition was higher than the neutral condition in channel Cz (*t*_(25)_ = 5.924, *p*_corrected_ < 0.001, *d* = 1.162). To differentiate emotional effects on LPP amplitude, a two-way repeated measures ANOVA including the emotion condition (positive, erotic, mutilation, and horror) and channel (Fz, Cz, and Pz) was performed. There were main effects of both channel and emotion condition with interaction between them (*F*_(1.164,27.937)_ = 7.019, *p* = 0.010, η_p_^2^ = 0.226; *F*_(3,72)_ = 5.598, *p* = 0.002, η_p_^2^ = 0.189; *F*_(2.753,63.070)_ = 3.998, *p* = 0.013, η_p_^2^ = 0.143). For the *post hoc* pairwise *t* test, both in channel Cz and Pz, *E_LPP, erotic_* and *E_LPP, horror_* were higher than *E_LPP, positive_* (Table 2). Increased LPP amplitude after seeing the erotic and horror pictures may reflect emotional arousal and regulation in these conditions.

### Facilitated or impeded attention and its prediction

**Figure 3.**
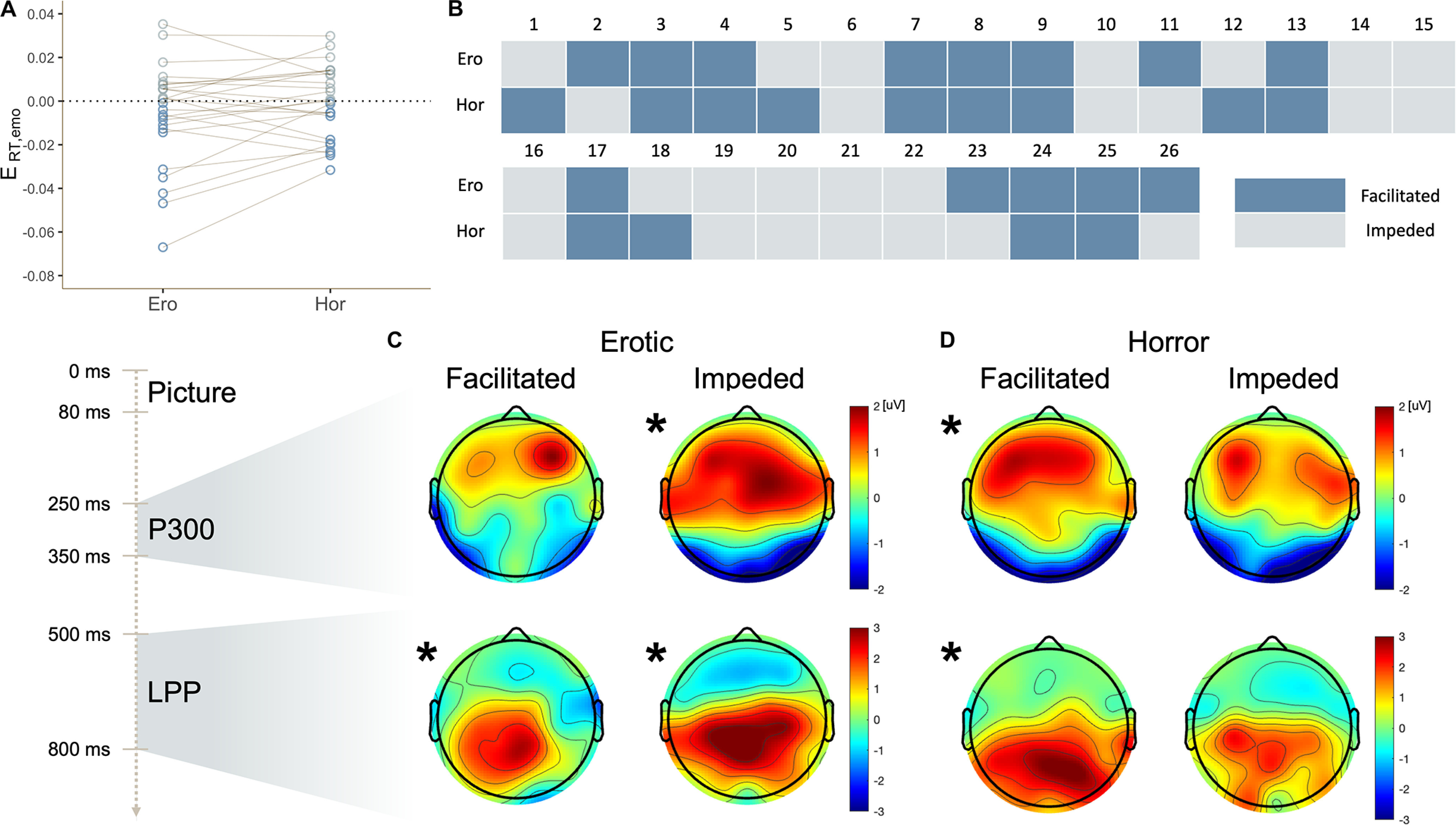
***A***, Individual differences in the direction of emotional effects on RT in the erotic and horror conditions. The dotted line indicates RT in the neutral condition. Each circle signifies individual RT difference scores *E_RT, emotion_* in the erotic and horror conditions. ***B***, Facilitated or impeded group placement. Based on individual emotional effects on RT, the 26 participants were divided into facilitated (*E_RT, emotion_* < 0) and impeded (*E_RT, emotion_* > 0) groups in each of the erotic and horror conditions. Individual group distribution is visualized using different shading. ***C***, ***D***, Topographical maps of ERP difference scores in the erotic condition (***C***) and horror condition (***D***). Black asterisks indicate that ERP amplitude in the erotic or horror condition was higher than the neutral condition. Top two topographic maps show *E_P300, erotic_* (***C***) and *E_P300, horror_* (***D***) and bottom maps show *E_LPP, erotic_* (***C***) and *E_LPP, horror_* (***D***). Topographic maps on the left side in each pair were from people whose attention was facilitated; and maps on the right side in each pair were from those whose attention was impeded.

Based on RT in the neutral condition (*E_RT, emotion_* = 0), participants were divided into two groups (facilitated vs impeded) in the erotic and horror conditions (Fig. 3*A*). Out of 26 participants, 13 were facilitated and the remaining 13 were disrupted in each condition (Fig. 3*B*). A nonparametric one-sample test (Wilcoxon signed-rank test) against 0 was conducted on *E_P300, emotion_* and *E_LPP, emotion_*. In the erotic condition (Fig. 3*C*), compared to the neutral condition, only participants showing impeded attention had higher P300 amplitude in response to the affective pictures in channels Fz and Cz (Z = 2.551, *p*_corrected_ = 0.022, *r* = 0.708; Z = 3.180, *p* = 0.003, *r* = 0.882). On the other hand, LPP amplitude increased in participants showing both facilitated and impeded attention in channels Cz and Pz (Z = 2.551, *p*_corrected_ = 0.011, *r* = 0.708; Z = 3.110, *p*_corrected_ = 0.003, *r* = 0.863; Z = 3.110; *p*_corrected_ = 0.003, *r* = 0.863; Z = 3.040, *p*_corrected_ = 0.003, *r* = 0.843). In the horror condition (Fig. 3*D*), compared to the neutral condition, only participants whose attention was facilitated showed higher P300 amplitude in channel Fz (Z = 2.341, *p*_corrected_ = 0.038, *r* = 0.649) and higher LPP amplitude in channel Pz (Z = 2.271, *p*_corrected_ = 0.046, *r* = 0.630). There were no differences in SCR, anxiety and depression scores, and gender between facilitated and impeded groups for both the erotic and horror conditions. To sum up, distraction in the erotic condition and facilitation in the horror condition showed distinct ERP profiles.

An identical experiment was conducted with an independent sample of 15 participants using a 32-channel EEG system (Neuroscan Grael and Curry 8 EEG software) for a partial replication of the original results. In the erotic condition, nine subjects were facilitated and six were disrupted (compared with the neutral condition); in the horror condition, eight were facilitated and seven were disrupted. Again, in the erotic condition, compared with the neutral condition, only participants showing impeded attention had higher P300 and LPP amplitude in channel Cz (Z = 1.992, *p* = 0.046, *r* = 0.813; Z = 2.201, *p* = 0.028, *r* = 0.899), while in the horror condition, participants whose attention was facilitated attention showed a tendency of higher P300 and LPP amplitude (Z = 1.820, *p* = 0.069, *r* = 0.644; Z = 1.820, *p* = 0.069, *r* = 0.644). Although slightly underpowered, these results suggest that our finding of the “cognotypes” in emotion-attention interaction are widespread and replicable.

Given the lack of a difference across channels in the repeated measures ANOVA and *post hoc* pairwise *t* tests, *E_P300, emotion_* in channels Fz, Cz, and Pz and *E_LPP, emotion_* in channels Cz and Pz were averaged. P300 and LPP were used as features for unimodal classification, and P300, LPP, and SCR were used as features for multimodal classification. In a unimodal classification based only on ERP measures, accuracy values from SVM, predicting whether attention would be facilitated or impeded were 53.0% (positive), 66.0% (erotic), 51.0% (mutilation), and 65.5% (horror). Mean 10-fold AUC values were 0.63 (positive), 0.73 (erotic), 0.48 (mutilation), and 0.76 (horror). On the other hand, in a multimodal classification based on ERP and SCR, accuracy values from SVM classifying whether attention would be facilitated or impeded were 56.17% (positive), 70.50% (erotic), 51.00% (mutilation), and 73.50% (horror). Mean 10-fold AUC values were 0.68 (positive), 0.74 (erotic), 0.46 (mutilation), and 0.81 (horror). The prediction accuracies of the direction of attentional effects in the erotic and horror conditions were significantly above chance level, 50% (*t*_(9)_ = 3.706, *p* = 0.005; *t*_(9)_ = 4.045, *p* = 0.003). Moreover, in a comparison of the unimodal and multimodal classification, overall accuracy and AUC were increased when SVM was performed with ERP and SCR (Table 3).

**Table 3:** Unimodal and multimodal classification accuracy and AUC for each emotional condition

Feature modality	Emotion	% Accuracy (SE)	AUC (SE)
Unimodal	Positive	53.0 (6.146)	0.631 (0.005)
	Erotic	66.0 (4.947)	0.761 (0.006)
	Mutilation	51.3 (6.125)	0.480 (0.046)
	Horror	65.5 (6.799)	0.762 (0.012)
Multimodal	Positive	56.2 (3.686)	0.686 (0.008)
	Erotic	70.5 (4.269)	0.742 (0.012)
	Mutilation	51.0 (6.902)	0.452 (0.047)
	Horror	73.5 (5.438)	0.817 (0.009)

SE: standard error; AUC: area under the ROC curve.

### Picture awareness ratings and recognition test

**Figure 4.**
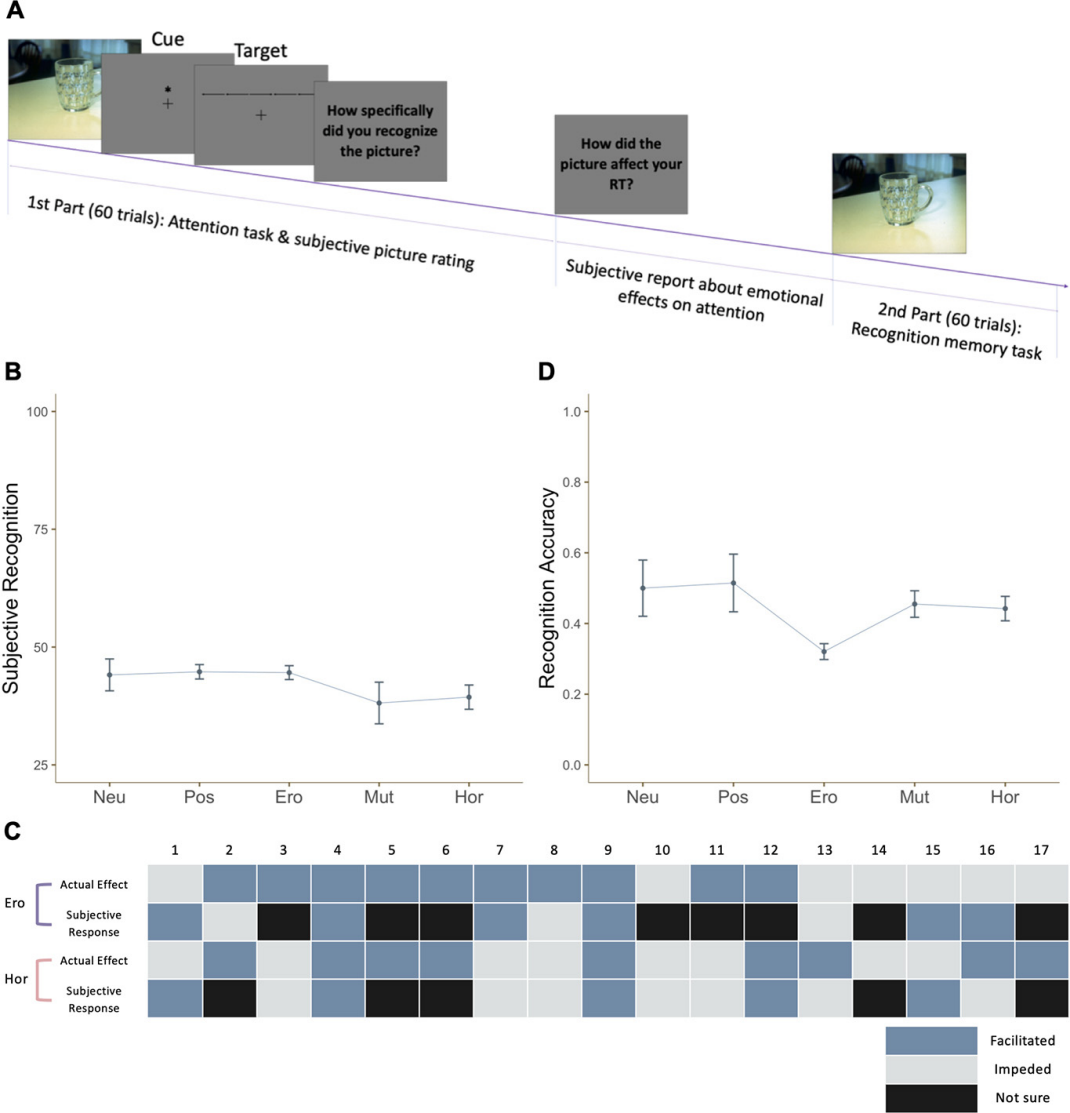
***A***, Task sequence. In the first part, an independent sample of 17 participants was asked to report their experience and awareness of the briefly-presented pictures after each trial of the task (60 trials total, 12 per picture category). Participants rated their perceived level of awareness of the presented picture after each trial. In the second part, half of the previously presented pictures and novel lure pictures in the same category were presented one by one in a recognition test in which subjects answered whether they had seen the picture. ***B***, Subjective awareness. 0: not aware; 25: color only; 50: emotional feeling; 75: partially-detailed recognition; and 100: perfect recognition. ***C***, Comparison of reported and actual effects of the pictures on attention. In the erotic and horror conditions, participants were also asked about how they felt their RT was influenced by the pictures (facilitated vs impeded). Their responses are shown alongside the actual attentional effects using different-colored shading; there was no significant correspondence between the two, meaning that participants were not aware of the effect that the pictures had on their subsequent attention. ***D***, Recognition memory accuracy. Participants were not able to distinguish the pictures they saw from lures in the same category, suggesting that while they were aware of the picture being flashed, they failed to process them in detail.

An additional supplementary experiment was conducted to see how participants processed the affective pictures in the present study. As in the original experiment, an affective picture was presented for 80 ms and followed immediately by a trial of the attention task (Fig. 4*A*). However, after each trial was completed, subjects were asked to answer several questions on the level of detail with which they perceived the picture and how much emotion was elicited by it. Five categories of pictures (neutral, positive, erotic, mutilation, and horror) were used, each consisting of 12 pictures from our original stimulus set. We found that participants were able to report the general gist of the pictures and their subjective feeling of arousal, but were not able to recall them in detail, regardless of picture type (Fig. 4*B*). After performing all 60 trials, they were also asked about how they thought their RT was influenced by the affective pictures (facilitated vs impeded); only four participants in the erotic condition and eight in the horror condition answered correctly (Fig. 4*C*). In the second part of the experiment, half of the previously presented pictures and novel lure pictures in the same category were presented one by one as a recognition test in which subjects answered whether or not they had seen the picture in the first part of the experiment. Recognition accuracies of all categories of pictures were not significantly higher than chance level, 50% (*t*_(16)_ = −0.436, *p*_corrected_ = 1, *d* = −0.106; *t*_(16)_ = 1.022, *p*_corrected_ = 0.966, *d* = 0.248; *t*_(16)_ = −10.661, *p*_corrected_ < 0.001, *d* = −2.586; *t*_(16)_ = −0.623, *p*_corrected_ = 1, *d* = −0.151; *t*_(16)_ = −1.578, *p*_corrected_ = 0.537, *d* = −0.383; Fig. 4*D*).

## Discussion

Through an investigation of individual differences, the present study clarifies previous mixed findings on whether emotional processing induces attentional assistance or interference. We found that neurophysiological responses to emotional processing in the erotic and horror conditions were reflected in ERP and SCR measures. Furthermore, it was possible to predict individual differences in the direction of subsequent attentional effects from the ERP and SCR measures, particularly in response to erotic and horror pictures.

### Salient emotion conditions

Significant increases in P300, and LPP were elicited and reliably predictable only in the erotic and horror conditions. Hajcak et al. (2010) pointed out that P300 amplitude is related to perceiving emotional significance and that LPP amplitude indicates emotional arousal and regulation. Moreover, the timing of the LPP is purported to reflect cognitive load. The higher LPP amplitude that we observed at 500–800 ms following the picture presentation can be additionally interpreted as the processing of emotional information with an additional cognitive demand, such as memory. Although this is a possibility, our supporting experiment showing participants’ failure in a subsequent recognition memory test suggests that higher memory processes were not involved in the timeframe we provided in our task (see Fig. 4). Therefore, rather than explicit emotional reappraisal or contextual memory processing, LPP may reflect a rapid emotional arousal regulation. According to this interpretation, the effects found in the erotic and horror conditions could reflect the perceptual significance of the stimuli (as indicated by the P300 response), followed by an automatic, rapid regulatory process before the attention task began (as indicated by the LPP response).

In past studies, explicit affective stimuli have been reported to elicit higher P300 or LPP amplitude than neutral stimuli, regardless of the specific emotional category (Naumann et al., 1992;  Lang et al., 1993, 1997, 1998; Bradley and Lang, 2000; Cuthbert et al., 2000; Bradley et al., 2001; Hajcak et al., 2010). Furthermore, the increase in P300 and LPP amplitudes after rapidly presented unpleasant stimuli were found to be weaker compared with the response to explicit stimuli (Ito and Cacioppo, 2000; Van Strien et al., 2010; Lin et al., 2018). Thus, our results suggest that only stimuli which are salient or potentially important may overcome the threshold for eliciting P300 or LPP. For instance, it is plausible that environmental stimuli related to mating opportunities or potentially harmful situations (as in the erotic and horror conditions) would be processed more quickly and effectively than others, albeit through different pathways of attentional modification.

In this sense, horror stimuli signifying a threat-related situational context might strongly elicit both cortico-limbic and sympathetic nervous system responses, reflected in the ERPs and SCR (Northoff et al., 2000; Carretié et al., 2004; Baumgartner et al., 2006). According to the Multiple Attention Gain Control (MAGiC) model (Pourtois et al., 2013), attentional processes can be enhanced indirectly by a mechanism of visual perception amplification triggered by emotion signals from the amygdala (that then consequently enhances performance in tasks requiring visual attention). The erotic condition, on the other hand, may modulate attention through a slightly different pathway that directly activates cortical attentional processes; one study, for instance, found that visual erotic stimuli activated the dorsolateral prefrontal cortex, which is known to play a crucial role in selective attention, and that this activation was sustained even after the stimulus disappeared (Leon-Carrion et al., 2007). This interpretation may explain why only people who responded significantly to the erotic stimulus showed a reduction in performance in the subsequent attention task.

### Individual differences in the direction of attentional effects

Past research reported mixed findings on the direction of the effect of emotion on attention (Schmeichel, 2007; Prause et al., 2008; Bocanegra and Zeelenberg, 2009, 2011; Rossignol et al., 2012; Brosch et al., 2013; Domınguez-Borras and Vuilleumier, 2013; Ortner et al., 2013; Pourtois et al., 2013). In our findings, attentional performance of some partipants was facilitated and that of others was impeded, depending on the erotic and horror conditions. This variation might be explained by differences in emotional response based on personal experiences and inclination toward the emotional stimuli (Zhang and Zhou, 2014; Matusz et al., 2015). For example, a past study reported that people who are afraid of snakes or spiders show a selectively higher LPP response to pictures of the particularly threatening objects than those who are not (Kolassa et al., 2005; Miltner et al., 2005).

In our study, LPP response to erotic pictures increased regardless of whether attention was facilitated or impeded, but P300 amplitude increased only for subjects who were impeded in the attention task. We interpret these results to mean that erotic pictures required emotion regulation in general, while they impeded subsequent attention only when people responded more strongly to them. In contrast, in the horror condition, both P300 and LPP amplitudes increased only for facilitated attention. It is possible that, although unpleasant stimuli are distracting in general, for individuals who are particularly responsive to the horror stimuli, they can assist subsequent attention (i.e., getting scared may enhance visual perception; Phelps et al., 2006; Bocanegra and Zeelenberg, 2009; Mobbs et al., 2009; Pourtois et al., 2013).

### Limitations

Our findings imply that there are certain types of people whose attentional effects of emotional processing can be dissociable depending on the emotion. In particular, their initial responses (P300) were highly indicative of the direction of attentional effects. However, we did not find a significant effect of the participants’ anxiety and depression scores, suggesting that there may be more complex factors contributing to individual sensitivity to specific types of emotional stimuli. Further investigations will delve into identifying these key factors to optimize our ability to predict and enhance cognitive performance at the individual level.

Furthermore, although we have speculated above on the distinct neural mechanisms in response to the horror and erotic stimuli, the difficulty in accessing signals directly from deep brain regions such as the amygdala using EEG makes it difficult for us to fully characterize these purported neural pathways underlying emotional processing and attention. A follow-up study using functional magnetic resonance imaging (fMRI) will make it possible to observe activity in deep brain structures in our task.

In conclusion, attentional effects of emotional processing may be unavoidable, as the fast and autonomic processing of stimuli may have evolved as an adaptive mechanism for subsequent behaviors. The present study provided a potential explanation for the directional effects of emotion on attention from the perspective of individual differences in emotional processing itself. Remarkably, these individual trends differed according to the category of emotion and were classifiable based on electrophysiological responses preceding the attention task. These findings may contribute to the development of personalized alerting or cognitive enhancement systems that cannot only optimize our performance in everyday life but also help prevent accidents and losses due to inattention.

## Synthesis

Reviewing Editor: Alexander Soutschek, Ludwig-Maximilians-Universitat Munchen Decisions are customarily a result of the Reviewing Editor and the peer reviewers coming together and discussing their recommendations until a consensus is reached. When revisions are invited, a fact-based synthesis statement explaining their decision and outlining what is needed to prepare a revision will be listed below. The following reviewer(s) agreed to reveal their identity: Ivan Grahek, Pawel Strozak.

The reviewers appreciate all the changes that were made by the authors and are convinced that the paper in its current form is significantly improved. It is worth mentioning that the authors have conducted additional experiments to support their initial findings and to deal with some of the concerns that were raised in the first round of reviews. Nonetheless, the reviewers point to some minor issues that still need to be addressed:

1. Abstract, lines 14-16: this is not very precise. The lack of large physiological response to erotic pictures in participants that were facilitated in this condition is only true for P300, but not for LPP (see also p. 17, lines 322-323). Please be more precise.

2. Still very little information is provided with regard to the measurement of skin conductance response (SCR). In the previous reviews, the reviewers had already asked for clarifying what exact measure of SCR activity was chosen, what was the time window for the measurement, how these data were analyzed and was there any decomposition of the SCR signal. In the revised version of the manuscript, only time window for SCR measurement is specified.

3. p. 9, line 170 - “(...) p-value of Emodality, emotion based on (...)”. It seems like there is a typographical error here.

4. Please explain supporting experiments in more detail. According to the extended data guidelines for eNeuro, the goal of including them “is not to report supplemental experiments or analyses that the authors are using to support the argument”. As these data are clearly aimed at supporting the results of the main experiment, they should be included in the main body of the manuscript, possibly in the “Results” section.

5. When reporting the results of recognition test of the additional experiment, please calculate and report the measure of subjects’ sensitivity (d prime). This is a better option than just the mere hit rate and can give us more insight into whether participants were really capable of discriminating old pictures from novel pictures.

## Author Response

We have clarified all points raised to the best of our ability. We hope you agree that these changes have made the manuscript suitable for publication. Thanks again for your consideration.

1. Abstract, lines 14-16: this is not very precise. The lack of large physiological response to erotic pictures in participants that were facilitated in this condition is only true for P300, but not for LPP (see also p. 17, lines 322-323). Please be more precise.

Thank you for pointing this out. We have rewritten those lines (lines 12 - 18 in revision) to be more precise:

We found that, in general, affective pictures induced higher electrophysiological responses compared to neutral pictures (P300 and late positive potential (LPP) in the erotic condition; P300, LPP, and SCR in the horror condition). In particular, individuals who showed a strong ERP response to the pictures were impeded (slower) in the erotic condition (only P300) and facilitated (faster) in the horror condition (both P300 and LPP). Those who did not show a significant ERP or SCR response to the pictures were facilitated in the erotic condition and impeded in the horror condition.

2. Still very little information is provided with regard to the measurement of skin conductance response (SCR). In the previous reviews, the reviewers had already asked for clarifying what exact measure of SCR activity was chosen, what was the time window for the measurement, how these data were analyzed and was there any decomposition of the SCR signal. In the revised version of the manuscript, only time window for SCR measurement is specified.

Thank you for these constructive comments. Because each trial of our task was quite short (about 4 seconds long) compared to the temporal dynamics of SCR, we implemented a block design in which subjects performed 24 trials of the same emotion condition in each block. We have clarified the measurement and analysis of SCR in the revised method section (lines 146 - 155):

SCR was measured by galvanic skin response to detect the release of sweat due to a change in arousal state (Montagu & Coles, 1966), using the Gazepoint biometrics package and software, with a constant voltage coupler (5 V) at a 60Hz sampling rate. Participants put their right index and middle fingers into the biometric hardware and were instructed to pull out their fingers between task blocks to prevent the physiological response from saturation. To calculate SCR for each picture, a high-pass FIR filter of 0.05Hz was applied (Matlab) to the entire dataset; then, maximum change from the baseline (average over 500 ms fixation period before picture onset) to the test trial (from picture onset to 500 ms before next trial) was extracted. The data was log-transformed to minimize skewed response and averaged for each block (for multimodal classification) and each emotional condition (for the remainder of the analysis).

3. p. 9, line 170 - “(...) p-value of Emodality, emotion based on (...)”. It seems like there is a typographical error here.

We have corrected this typographical error in the revision.

4. Please explain supporting experiments in more detail. According to the extended data guidelines for eNeuro, the goal of including them “is not to report supplemental experiments or analyses that the authors are using to support the argument”. As these data are clearly aimed at supporting the results of the main experiment, they should be included in the main body of the manuscript, possibly in the “Results” section.

We have now moved the Extended Data 1 to the Results section (New Figure 4 and lines 292 - 307). The replication results in Extended Data 2 have been moved as text to the Result section (lines 267 - 275).

5. When reporting the results of recognition test of the additional experiment, please calculate and report the measure of subjects’ sensitivity (d prime). This is a better option than just the mere hit rate and can give us more insight into whether participants were really capable of discriminating old pictures from novel pictures.

Thank you. We have included effect size values in the revision.
